# Mobile Breast Cancer e-Support Program for Chinese Women With Breast Cancer Undergoing Chemotherapy (Part 2): Multicenter Randomized Controlled Trial

**DOI:** 10.2196/mhealth.9438

**Published:** 2018-04-30

**Authors:** Jiemin Zhu, Lyn Ebert, Xiangyu Liu, Di Wei, Sally Wai-Chi Chan

**Affiliations:** ^1^ Nursing Department Medical School Xiamen University Xiamen China; ^2^ School of Nursing and Midwifery Faculty of Health and Medicine University of Newcastle Newcastle Australia; ^3^ Hunan Cancer Hospital Xiangya School of Medicine Central South University Changsha China

**Keywords:** breast cancer, chemotherapy, mobile app, self-efficacy, social support

## Abstract

**Background:**

Women undergoing chemotherapy for the treatment of breast cancer have frequently reported unmet supportive care needs. Moreover, easily accessible and innovative support is lacking.

**Objective:**

The purpose of this trial was to determine the effectiveness of an app-based breast cancer e-support program to address women’s self-efficacy (primary outcome), social support, symptom distress, quality of life, anxiety, and depression. Secondary objectives included exploring the association between women’s health outcomes and the breast cancer e-support usage data.

**Methods:**

A multicenter, single-blinded, randomized controlled trial was conducted. A total of 114 women with breast cancer, who were commencing chemotherapy and were able to access internet through a mobile phone, were recruited in the clinics from 2 university-affiliated hospitals in China. Women were randomized either to the intervention group (n=57) receiving breast cancer e-support plus care as usual or the control group (n=57) receiving care as usual alone. The health care team and research assistants collecting data were blinded to the women’s group allocation. Bandura’s self-efficacy theory and the social exchange theory guided the development of the breast cancer e-support program, which has 4 components: (1) a Learning forum, (2) a Discussion forum, (3) an Ask-the-Expert forum, and (4) a Personal Stories forum. Moderated by an experienced health care professional, the breast cancer e-support program supported women for 12 weeks covering 4 cycles of chemotherapy. Health outcomes were self-assessed through paper questionnaires in clinics at baseline before randomization (T0), after 3 (T1), and 6 months (T2) of follow-ups.

**Results:**

Fifty-five participants in the intervention group and 49 in the control group completed the follow-up assessments (response rate: 91.2%). During the 12-week intervention, the log-in frequency ranged from 0 to 774 times (mean 54.7; SD 131.4; median 11; interquartile range, IQR 5-27), and the total usage duration ranged from 0 to 9371 min (mean 1072.3; SD 2359.5; median 100; IQR 27-279). Repeated measures multivariate analysis of covariance (intention-to-treat) found that breast cancer e-support + care as usual participants had significant better health outcomes at 3 months regarding self-efficacy (21.05; 95% CI 1.87-40.22; *P*=.03; *d*=0.53), symptom interference (−0.73; 95% CI −1.35 to −.11; *P*=.02; *d*=−0.51), and quality of life (6.64; 95% CI 0.77-12.50; *P*=.03, *d*=0.46) but not regarding social support, symptom severity, anxiety, and depression compared with care as usual participants. These beneficial effects were not sustained at 6 months. Spearman rank-order correlation showed that the breast cancer e-support usage duration was positively correlated with self-efficacy (*r*=.290, *P*=.03), social support (*r*=.320, *P*=.02), and quality of life (*r*=.273, *P*=.04) at 3 months.

**Conclusions:**

The breast cancer e-support program demonstrated its potential as an effective and easily accessible intervention to promote women’s self-efficacy, symptom interference, and quality of life during chemotherapy.

**Trial Registration:**

Australian New Zealand Clinical Trials Registry (ANZCTR): ACTRN12616000639426; www.ANZCTR.org.au/ACTRN12616000639426.aspx (Archived by Webcite at http://www.webcitation.org/6v1n9hGZq)

## Introduction

Breast cancer is a major public health problem worldwide. In China, breast cancer is the most frequently diagnosed cancer for women, and approximately 81.4% of women with invasive breast cancer receive chemotherapy [1]. However, chemotherapy results in side effects such as pain, fatigue, and sleep disturbance, which adversely affect women’s quality of life (QoL) and psychological well-being [2]. These women frequently report unmet supportive care needs [3]. To better support women with breast cancer undergoing chemotherapy, health promotion efforts must provide appropriate symptom management strategies, as well as build a sense of self-efficacy and social support to initiate and maintain such desired strategies [4]. With advanced technology, mobile apps provide a promising platform in ways that allow women with breast cancer to acquire knowledge and interact with peers or health care professionals when and where needed [5].

In 2017, there were approximately 1.35 billion mobile phone users in China, accounting for 89% of the Chinese population [6]. It should be possible to use apps to promote quality health care through a robust and easily accessible program. However, there remains a paucity of randomized controlled trials (RCTs) to evaluate the effectiveness of app-based programs targeting women with breast cancer undergoing chemotherapy [7]. To date, most app-based programs including women with breast cancer have not been chemotherapy specific [8], or breast cancer specific [9]. Furthermore, women’s usage of eHealth interventions and their relationship with effectiveness has rarely been reported in trials [10].

We developed the app-based, interactive breast cancer e-support (BCS) program (ACTRN12616000639426) [11] under the guidance of the incorporation of Bandura’s self-efficacy theory [12] and social exchange theory [13]. The BCS theoretical framework has been demonstrated to be useful in the design of a psychoeducational program to optimize patients’ health outcomes [14]. The purpose of this trial was to determine the effectiveness of BCS regarding women’s health outcomes. Secondary objectives included exploring the association between women’s health outcomes and the BCS usage data. A descriptive qualitative study was employed in Part 1 of this study to explore participant perceptions of the BCS for those in the intervention arm [15]. We hypothesized that BCS+CAU participants would show significant better health outcomes in self-efficacy, social support, symptom management, QoL, anxiety, and depression across time compared with CAU participants. We also hypothesized that, as more women used the BCS program, better health outcomes would be achieved. To the best of our knowledge, this is the first study of its kind in China to evaluate app effectiveness for women with breast cancer undergoing chemotherapy.

## Methods

### Study Design and Participants

The BCS study protocol was published in *BMC Cancer* [11]. A multicenter, single-blinded, parallel RCT was used to evaluate the effectiveness of BCS. Women were eligible to participate if they were diagnosed with any stage of breast cancer within the prior 3 to 8 weeks, were able to access the internet through the mobile phone, were able to read and write Mandarin, and were commencing chemotherapy. Women were excluded if they had concurrent major physical illnesses or chronic mental health conditions.

The study was conducted between May 2016 and February 2017 at two university-affiliated hospitals in China. Ethics approvals were granted from the Institutional Review Board of Xiamen University affiliated Zhong Shan Hospital (ZSH) and Central South University affiliated Hunan Cancer Hospital (HCH) in China and the University of Newcastle in Australia. The clinicians introduced the BCS program to eligible women in the oncology clinics, and the researchers (JZ and DW) met interested women, confirmed their eligibility, and obtained their consent forms. After baseline data collection, the researchers (JZ and DW) randomly allocated women to BCS program plus care as usual (BCS+CAU) or CAU-alone group with allocation ratio as 1:1 and provided 30 min of program training for BCS+CAU participants before their first cycle of chemotherapy. The research assistants (RAs) collected data at baseline (T0), at 3 months (T1), and at 6 months (T2) of medical follow-ups with self-assessed paper questionnaires in the clinics. These time frames were chosen because greatest benefits of internet-based intervention were documented within 3 months [16], and some benefits might be sustained 6 months later [17]. Women were provided with a small gift (approximately US $5) when they returned their questionnaires.

### Intervention

The process of BCS development was published in *Technology and Health Care* [18]. User-centric design was applied in BCS development, and the perceived ease of using the BCS program was assessed [18]. The researchers (JZ and DW) helped BCS+CAU participants to download the app into their mobile phones and to register the BCS program. After approval by the first author from the app background thread, a unique username was generated with automated passport (changeable later). BCS+CAU participants did not need to pay for BCS access, and the usernames expired 12 weeks after activation.

**Figure 1:**
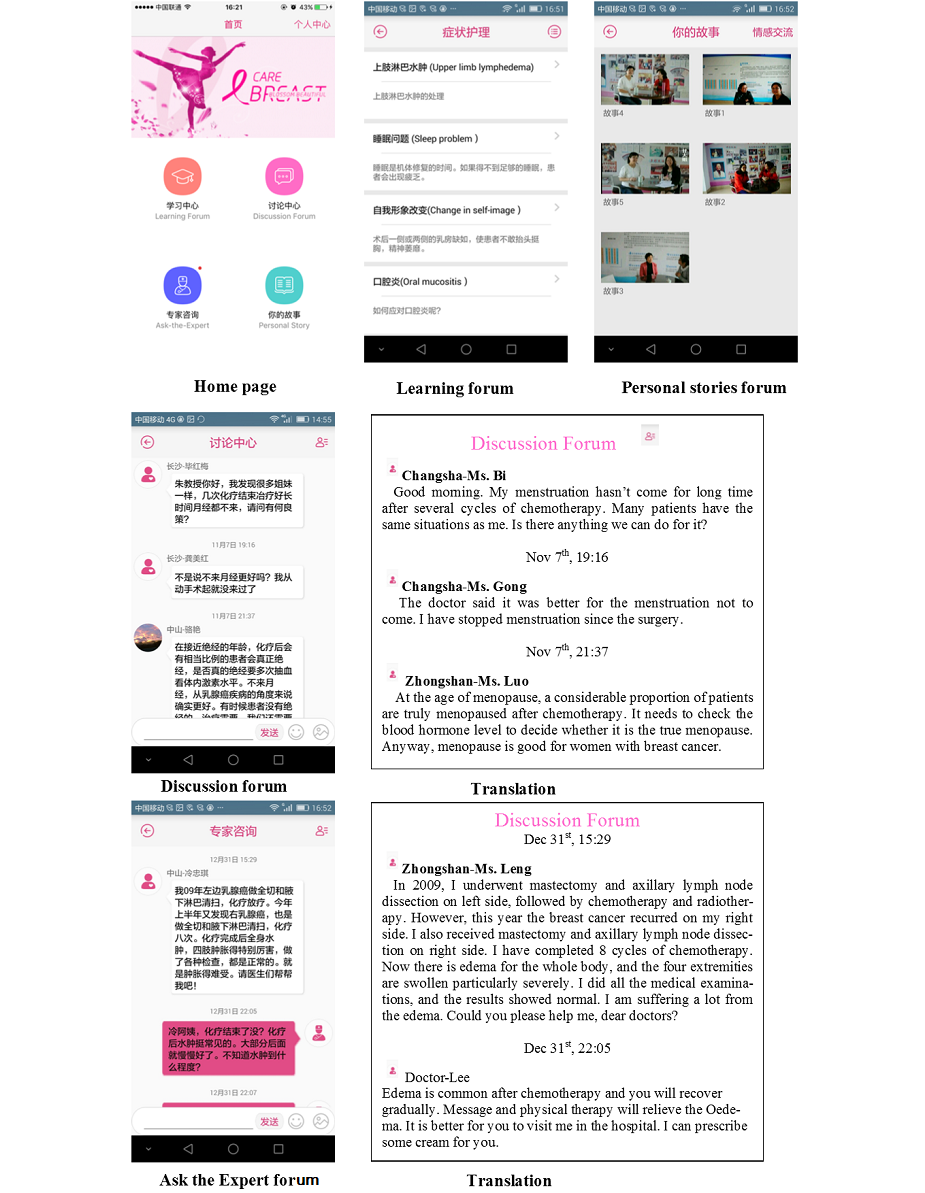
Screenshots of the breast cancer e-support (BCS) program home page and the 4s forums.

Because 4 cycles of chemotherapy (3 weeks/cycle) are the minimum recommended standard [1], BCS program supported women for 12 weeks covering from the beginning of the 1st cycle to the end of the 4th cycle of chemotherapy. The BCS program (Figure 1) included 4 components: (1) a Learning forum; (2) a Discussion forum; (3) an Ask-the-Expert forum; and (4) a Personal Stories forum [11]. On the basis of Bandura’s self-efficacy theory (direct mastery experiences, vicarious experiences, verbal persuasion, and arousal state) [12], the Learning forum provided knowledge related to breast cancer and symptom management strategies to address the women’s direct mastery experiences. All knowledge was evidence-based and validated by multidisciplinary Chinese oncology professionals. The Discussion forum and Ask-the-Expert forum offered opportunities for women to interact with peers and health care professionals where verbal persuasion and modification of the women’s perceptions of arousal states occurred. The Personal Stories forum involved 5 video-recorded encouraging stories to enhance the women’s vicarious experiences. Guided by the social exchange theory (structural and functional support) [13], the Discussion forum and Ask-the-Expert forum increased the women’s structural social networks, and the interaction within these 2 forums conveyed various functional support.

On the basis of the questions and concerns put forward in the BCS program, the Learning forum was updated with new knowledge every 2 weeks. The moderator, an experienced health care professional, moderated the Discussion forum by reading all messages daily and providing expert advice if requested. To protect the women’s privacy, access to the questions and response in the Ask-the-Expert forum were restricted to the individual posing the question and health care professionals. Eight doctors from the participating hospitals joined the BCS program. The moderator sent reminder message to the corresponding doctors with incoming questions, and the doctors answered women’s questions in the Ask-the-Expert forum within 24 hours. With the women’s permission, some valuable questions and answers, which might be interesting for others, were added to the Discussion forum to facilitate communication. Technical assistance was available to the women during the workday. It was up to women how often and how long they made use of the BCS program.

### Comparator

Women receiving CAU alone did not have BCS access. For both conditions, CAU consisted of health supportive care while receiving chemotherapy as an inpatient. There were no restrictions in both groups in terms of performing other internet searches for information or social support.

### Outcomes

Women self-reported sociodemographic and clinical variables at T0. The medical records were checked if doubts existed regarding the clinical variables.

The primary outcome was self-efficacy at 3 months comparing the intervention and control arms. Self-efficacy was assessed using the Chinese version of the Stanford Inventory of Cancer Patient Adjustment (SICPA), which is a 38-item instrument to evaluate women’s belief in their ability to manage problems related to cancer [19]. The total score of SICPA ranges from 0 to 380, with higher total scores indicating higher level of self-efficacy. The baseline internal consistency of SICPA for this study was good (Cronbach alpha=.87).

Secondary outcomes measured the women’s social support, symptom distress, QoL, and anxiety and depression. Social support was assessed using the Chinese version of the Multidimensional Scale of Perceived Social Support (MSPSS), which is a 12-item self-report instrument to evaluate women’s perception of support [20]. The item score ranges from 1 to 7, with a higher mean score indicating better social support. In this study, the baseline internal consistency of the MSPSS was .89.

Symptom distress was assessed using the Chinese version of the MD Anderson Symptom Inventory, which consists of a 13-item symptom severity subscale to measure the severity of each symptom and a 6-item symptom interference subscale to evaluate the extent to which the symptoms interfere with the patients’ daily life [21]. The item score ranges from 1 to 10, with a higher mean score indicating severer symptom distress. In this study, the baseline internal consistency for symptom severity and symptom interference were .77 and .84, respectively.

QoL was assessed with a Chinese version of the Functional Assessment of Cancer Treatment-B (FACT-B), which is a 37-item instrument to evaluate the impact of breast cancer and its chemotherapy on dimension of QoL [22]. The total score of FACT-B ranges from 0 to 148, with higher total scores indicating better QoL. FACT-B had good baseline internal consistency in this study (Cronbach alpha=.77).

Anxiety and depression were assessed using the Chinese version of the Hospital Anxiety and Depression Scale, which consists of a 7-item anxiety subscale and a 7-item depression subscale [23]. The total score of each subscale ranges from 0 to 21, with higher total scores indicates greater anxiety or depression. In this study, the baseline internal consistencies were .81 and .73 for the anxiety and depression subscales, respectively.

Twelve weeks’ usage data, including log-in frequency and usage duration of the whole BCS program, were tracked in the app’s statistics module of background thread on individual basis. Log-in frequency was recorded as the number of times a participant logged into the app during 12 weeks. The total usage duration was recorded as the sum of all time in minutes between logging in and logging out. If women forgot logging out of the app, the app ran as the background operation mode no matter women were surfing on other websites or the mobile phones were in standby modes. App running as the background operation mode was regarded as app being logged out in the app’s statistics module when recording the usage duration.

### Random Assignments and Masking

Women with breast cancer were randomly assigned to BCS+CAU or CAU alone with an allocation ratio as 1:1. For each hospital, a permuted block randomized design was used with Research Randomizer (Urbaniak and Plous) [24]. A variety of randomly selected block sizes of 4, 6, and 8 ensured blinded allocation. The health care team and RAs collecting data were blinded to the women’s group allocation.

### Sample Size Calculation

The study sample size was determined by the primary outcome of self-efficacy, with the standardized effect size of 0.60 reported in a previous psychosocial trial [25]. A sample of 108 participants (54 participants per group) was needed to detect an effect size of at least 0.60, with 80% power, two-sided *P*<.05, and 20% attrition. A dropout rate ranging from 10% to 20% was reported in previous studies involving an app-based study [9,26]. Finally, the recruitment numbered 114 participants in total (57 participants per group).

### Statistical Analysis

IBM SPSS Statistics 22.0 (IBM Corp, New York, USA) was used to analyze the data [27]. Intention-to-treat analysis with the last observation carried forward was applied to account for missing data. All baseline demographic characteristics, clinical variables, and baseline outcomes were compared using independent samples *t* test for continuous variables and chi-square test or Fisher exact test for categorical variables between the randomized assigned groups, as well as between participants who completed all follow-ups and who dropped out. The effectiveness of intervention on the primary and secondary outcomes was tested using repeated measures multivariate analysis of covariance with a group as a between-subject factor, time as a within-subject factor, and the interaction between group and time, adjusted by baseline corresponding outcomes. Because the women were randomly assigned at each hospital, the hospital site was not included as a random effect. The adjusted mean difference (95% CI) between groups at each of the following-up points are reported, with the adjusted mean (SDs), significance level, and effect size (Cohen *d*). The adjusted means and pooled SD were used to calculate the effect size Cohen *d* for independent groups. With the caveat that only for women in the intervention group, the mean (SD), median, interquartile range (IQR), and maximum were used to describe log-in frequency and usage duration of the BCS program. Due to the highly skewed nature of the BCS usage data, Spearman rank-order correlation was calculated between the women’s BCS usage data and unadjusted outcome variables at three time points. *P*<.05 was considered statistically significant.

## Results

### Participant Characteristics

**Figure 2:**
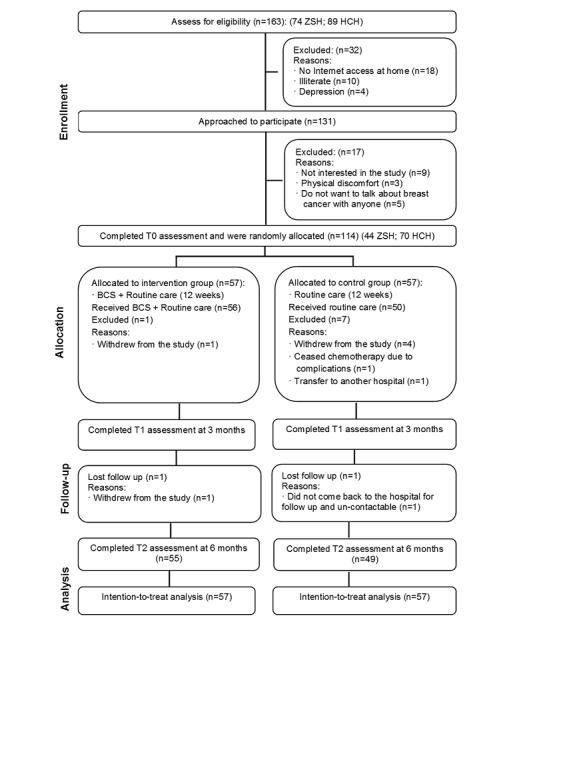
Consolidated Standard of Reporting Trials (CONSORT) diagram of Breast Cancer e-Support program (BCS) program. HCH: Central South University affiliated Hunan Cancer Hospital; ZSH: Xiamen University affiliated Zhong Shan Hospital.

Between May 2016 and August 2016, 163 women were assessed for eligibility: 32 women (19.6%) were ineligible, 17 women (10.4%) refused, and 114 women (69.9%) underwent random assignment. Of the 114 women randomly assigned, 44 women (38.6%) were recruited from ZSH, and 70 women (61.4%) were recruited from HCH. Data collection was finalized in February 2017. Figure 2 presents the Consolidated Standard of Reporting Trials flowchart [28].

The two groups were comparable at baseline regarding demographic, clinical-related, and outcome measures (Table 1). There were more participants with missing data at T1 among the CAU participants (n=7) than among the BCS+CAU participants (n=1), but the difference was not significant (*P*=.06). Baseline variables in Table 1 did not show a significant difference between participants who completed all follow-ups (n=104) and who dropped out (n=10). A total of 96% of BCS+CAU participants (n=55) and 86% of CAU participants (n=49) completed the follow-up assessments.

### Effectiveness of the Breast Cancer e-Support Program

Regarding the primary outcomes, the women’s self-efficacy in both groups was reduced after chemotherapy began. Adjusted for the baseline self-efficacy, the decrease in self-efficacy at T1 was significantly less for BCS+CAU participants than for CAU participants, with a medium effect size (*P*=.03; *d*=0.53; adjusted mean difference=21.05; 95% CI 1.87-40.22; Table 2).

**Figure 3:**
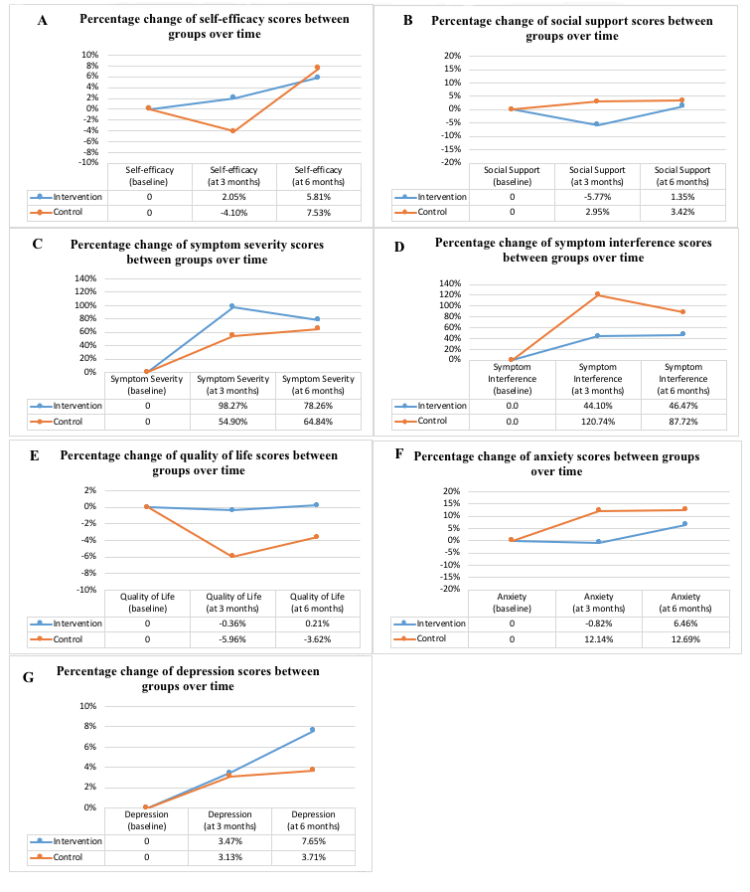
Mean percentage changes of health outcomes from baseline to posttests (n=114). Score at 3 months percentage change=(score at 3 months-score at baseline)/score at baseline; Score at 6 months percentage change=(score at 6 months-score at baseline)/score at baseline.

Regarding the secondary outcomes, both symptom severity and symptom interference increased from T0 to T1. Adjusted for baseline symptom interference, the increase in symptom interference at T1 was significantly less for BCS+CAU participants than for CAU participants, with a medium effect size (*P*=.02; *d*=−0.51; adjusted mean difference=−.73; 95% CI −1.35 to −.11; Table 2). No such difference in symptom severity was found. The QoL declined following the commencement of chemotherapy. Controlled for baseline QoL, the drop in QoL for BCS+CAU participants was significantly less than that for CAU participants, with a small to medium effect size (*P*=.03, *d*=0.46; adjusted mean difference=6.64; 95% CI 0.77-12.50; Table 2). There was no significant group difference for social support, anxiety, and depression from T0 to T1. At the 6-month follow-up, our intervention did not lead to significant improvement in any health outcomes from T1 to T2. Figure 3 presents a graphical representation of the mean percentage change in health outcomes. The hypothesis that the intervention could enhance health outcomes was partially supported.

### Association Between the Breast Cancer e-Support Usage Data and Health Outcomes

BCS usage varied considerably. During the 12-week intervention, the log-in frequency ranged from 0 to 774 times (mean 54.7; SD 131.4; median 11; IQR 5-27), and the total usage duration ranged from 0 to 9371 min (mean 1072.3; SD 2359.5; median 100; IQR 27-279). Two BCS+CAU participants never logged into the BCS program. The association between log-in frequency and outcomes variables was not found in this study.

BCS usage duration was correlated with different health outcomes at three time points. At T0, self-efficacy (*r*=.439, *P*=.001) and QoL (*r*=.313, *P*=.02) showed a positive correlation with the BCS usage duration, whereas symptom severity (*r*=−.297, *P*=.03) was inversely related to the women’s BCS usage duration. At T1, self-efficacy (*r*=.290, *P*=.03), social support (*r*=.320, *P*=.02), and QoL (*r*=.273, *P*=.04) were positively related to the BCS usage duration. At T2, self-efficacy was still correlated with the BCS usage duration (*r*=.329, *P*=.01), whereas anxiety was inversely correlated with the BCS usage duration at T2 (*r*=−.300, *P*=.03).

**Table 1:** Comparison of demographic/clinical characteristics and baseline outcomes between the groups (n=114).

Demographic/clinical characteristics and baseline outcomes^a^		Total (N=114)	BCS^b^+CAU^c^ participants (N=57)	CAU participants (N=57)
Age in years, mean (SD)		47.2 (8.3)	46.2 (8.5)	48.2 (8.1)
**Marital status, n (%)**				
	Married	111 (97.4)	57 (100)	54 (95)
	Single	2 (1.8)	0 (0)	2 (1)
	Divorce	1 (0.9)	0 (0)	1 (1)
**Education level, n (%)**				
	No education	17 (14.9)	8 (14)	9 (16)
	Elementary school	31 (27.2)	13 (23)	18 (32)
	Junior middle school	33 (28.9)	16 (28)	17 (30)
	High school	21 (18.4)	12 (21)	9 (16)
	University or above	12 (10.5)	8 (14)	4 (7)
**Monthly family income (USD)^d^, n (%)**				
	≤148	22 (19.3)	14 (25)	8 (14)
	149-442	60 (52.6)	24 (42)	36 (63)
	443-738	17 (14.9)	10 (18)	7 (12)
	≥739	14 (12.3)	8 (14)	6 (11)
	Missing data	1 (0.9)	1 (2)	0 (0.0)
**Currently employment^d^, n (%)**				
	Yes	19 (16.7)	10 (18)	9 (16)
	No	86 (75.4)	44 (77)	42 (74)
	Missing data	9 (7.9)	3 (5)	6 (11)
Body mass index (kg/m^2^), mean (SD)		23.4 (2.9)	23.0 (2.6)	23.7 (3.2)
**Cancer stage, n (%)**				
	1	21 (18.4)	9 (16)	12 (21)
	2	49 (43.0)	28 (49)	21 (37)
	3	42 (36.8)	19 (33)	23 (40)
	4	2 (1.8)	1 (2)	1 (2)
**Surgery, n (%)**				
	Breast conserving surgery	5 (4.4)	3 (5)	2 (4)
	Mastectomy	97 (85.1)	45 (79)	52 (91)
	Others	12 (10.5)	9 (16)	3 (5)
**Comorbidity, n (%)**				
	Yes	3 (2.6)	1 (2)	2 (4)
	No	111 (97.4)	56 (98)	55 (96)
**Complication, n (%)**				
	Yes	1 (0.9)	0 (0)	1 (2)
	No	113 (99.1)	57 (100)	56 (98)
**Cycles of chemotherapy, n (%)**				
	Four cycles	18 (15.7)	9 (16)	9 (16)
	Six cycles	27 (23.7)	11 (19)	16 (28)
	Eight cycles	69 (60.5)	37 (65)	32 (56)
**Chemotherapy regimen, n (%)**				
	Cyclophosphamide+Epirubicin+Docetaxel	47 (41.2)	25 (44)	22 (39)
	Docetaxel+Cyclophosphamide+Herceptin	18 (15.8)	9 (16)	9 (16)
	Theprubicine+Cyclophosphamide+Docetaxel+Herceptin	17 (14.9)	8 (14)	9 (16)
	Liposomal doxorubicin or Pharmorubicin+Cyclophosphamide	14 (12.3)	5 (9)	9 (16)
	Herceptin	8 (7.0)	4 (7)	4 (7)
	Vinorelbine+Cisplatin or Lobaplatin	7 (6.1)	4 (7)	3 (5)
	Others	3 (2.6)	2 (4)	1 (2)
**Health outcomes, mean (SD)**				
	Self-efficacy	224.7 (59.2)	235.3 (64.6)	214.1 (51.7)
	Social support	5.5 (0.7)	5.6 (0.7)	5.4 (0.8)
	Symptom severity	3.5 (2.0)	3.3 (2.0)	3.7 (2.0)
	Symptom interference	3.1 (1.9)	2.8 (1.8)	3.3 (2.1)
	Quality of life	92.8 (18.4)	94.6 (19.5)	90.9 (17.3)
	Anxiety	9.9 (2.4)	10.3 (2.4)	9.5 (2.3)
	Depression	12.6 (2.2)	12.6 (2.0)	12.6 (2.4)

^a^No significant difference were found between two groups (*P*>.05). *P* values were calculated using independent samples *t* test for continuous variables and chi-square tests or Fisher exact test for categorical variables.

^b^BCS: breast cancer e-support program.

^b^CAU: care as usual.

^d^Missing data present.

**Table 2:** Effect of breast cancer e-support (BCS) program (intention-to-treat analysis) on primary and secondary outcomes at 3 months (T1) and 6 months (T2); N=114.

Treatment effect			Mean (SD)		Adjusted mean difference (95% CI)	*P* value^a^	Effect size (Cohen *d*)
			BCS+CAU^b^ participants (n=57)	CAU participants (n=57)						
**Primary outcomes**							
	**Self-efficacy (SICPA^c^) [19]**						
		T1	227.12 (66.80)	197.07 (43.44)	21.05 (1.87 to 40.22)	*.03*	0.53
		T2	232.09 (69.01)	220.91 (56.32)	3.40 (−19.05 to 25.86)	.76	0.18
**Secondary outcomes**							
	**Social support (MSPSS^d^) [20]**						
		T1	5.24 (1.00)	5.47 (2.75)	−0.39 (−1.15 to 0.38)	.32	−0.11
		T2	5.62 (.65)	5.42 (.80)	0.14 (−0.13 to 0.41)	.31	0.27
	**Symptom severity (MDASI^e^) [21]**						
		T1	3.79 (1.81)	4.17 (1.71)	−0.21 (−0.72 to 0.31)	.42	−0.22
		T2	3.67 (2.21)	4.30 (1.89)	−0.26 (−0.88 to 0.36)	.41	−0.31
	**Symptom interference (MDASI) [21]**						
		T1	2.99 (1.78)	3.93 (1.91)	−0.73 (−1.35 to −0.11)	*.02*	0.51
		T2	3.11 (2.01)	3.84 (1.95)	−0.57 (−1.27 to 0.13)	.11	−0.37
	**Quality of life (FACT-B^f^) [22]**						
		T1	92.87 (21.39)	84.09 (15.99)	6.64 (0.77 to 12.50)	*.03*	0.46
		T2	92.16 (21.24)	85.66 (15.58)	5.23 (−1.34 to 11.80)	.12	0.35
	**Anxiety (HADS^g^) [23]**						
		T1	9.93 (2.72)	10.28 (2.46)	−0.77 (−1.62 to 0.08)	.07	−0.14
		T2	10.58 (2.87)	10.26 (2.39)	−0.05 (−0.96 to 0.86)	.92	0.12
	**Depression (HADS) [23]**						
		T1	12.75 (1.57)	12.58 (2.15)	0.17 (−0.52 to 0.87)	.62	0.09
		T2	13.28 (2.02)	12.65 (2.53)	0.63 (−0.20 to 1.45)	.14	0.28

^a^All *P* values were calculated using an analysis of covariance with adjustment for baseline value of the corresponding questionnaire.

^b^CAU: care as usual.

^c^SICPA: Stanford Inventory of Cancer Patient Adjustment.

^d^MSPSS: Multidimensional Scale of Perceived Social Support.

^e^MDASI: MD Anderson Symptom Inventory.

^f^FACT-B: Functional Assessment of Cancer Treatment-B.

^g^HADS: Hospital Anxiety and Depression Scale.

## Discussion

### Principal Findings

The strength of this study included health-focused theoretical underpinnings that support the design of the BCS program and the study’s methodological rigor in data collection and analysis. This study found that, when women are in the midst of early struggle with breast cancer and chemotherapy, 12-week access to BCS program plus CAU resulted in significant better health outcomes regarding self-efficacy, symptom interference, and QoL compared with CAU alone at 3 months. However, these beneficial effects were not sustained at 6 months. Access to BCS did not influence social support, symptom severity, anxiety, and depression at follow-ups. The BCS usage duration was positively correlated with self-efficacy, social support, and QoL at 3 months.

BCS participants reported significantly better self-efficacy at 3 months compared with control participants. In addition, a positive relationship was found between self-efficacy and the BCS usage duration at baseline, 3 months, and 6 months of follow-ups. Self-efficacy determines whether the women would initiate the actions, how much effort they exerted, and how long they sustained the effort when encountered with obstacles [12]. Consistent with prior research [14,25], we demonstrated that the self-efficacy theory and social exchange theory are usable in guiding the development of an app-based program to enhance self-efficacy.

BCS+CAU participants showed better QoL at 3 months. Furthermore, BCS usage duration was positive related with QoL at baseline and 3 months. Similarly, Gustafson et al [29] reported that the Comprehensive Health Enhancement Support System (CHESS) had a positive impact on QoL for women with breast cancer. CHESS is a computer-based program involving an information module, a communication module, and an interactive coaching module [16], which are, currently, easy to install and use through apps. Our BCS program indicated that the app-based BCS program could provide comparative functionality and achieve similar effectiveness as computer-based programs [29], whereas women could enjoy the advantage of convenience and easy access of apps.

This study achieved a significant group difference in symptom distress only for the subscale of symptom interference, not for the subscale of symptom severity, at 3 months. Decreased symptom distress is a critical indicator of successful health support [30]. BCS program may modify women’s interpretation of the extent to which symptoms interfered with their daily lives. In this study, symptom severity was inversely correlated with BCS usage duration at baseline. Some women might have experienced high levels of symptoms such as pain or fatigue that hindered their BCS engagement, potentially diluting the results. Future app-based studies might involve caregivers using the app to support the patients when the patients are experiencing severe symptoms.

BCS program did not significantly change social support relative to the effect of CAU alone at 3 months. In China, there are many existing popular mobile phone–based chat platforms, such as Webchat and QQ, which women in both groups were more familiar with and used for seeking social support, thus potentially competing for the impact of BCS program on social support. However, among BCS+CAU participants, our study found that women’s BCS usage duration was positively associated with the perceived social support at 3 months, indicating that the longer women used the BCS program, the higher women perceived social support. The BCS superiority to other online chat platform is the credibility of information provided and medical consultation from experts, which should be addressed to promote engagement with BCS.

In addition, the study found that the BCS program did not significantly reduce the BCS+CAU participants’ anxiety and depression at 3 months. Access to a wide variety of knowledge related to breast cancer and chemotherapy may not relieve the women’s anxiety and depression [31]. Moreover, literature shows inconsistent findings regarding the effects of eHealth on anxiety and depression for cancer patients [32], which needs to be addressed in the future research.

This study found no long-term effects for women at 6 months. This may be because women could access the BCS program for 12 weeks only, and the BCS program may produce little residual advantage at 6 months. However, the physical and psychosocial symptoms may persist for 12 months or even longer after the completion of the chemotherapy [33]. Thus, allowing women to retain BCS access longer may have revealed different outcomes at 6 months. Moreover, the majority of participating women had completed chemotherapy and were experiencing physical, psychological, and social recovery at 6 months. It is possible that BCS program focuses on chemotherapy support and does not include sufficient knowledge for adjustment after completion of the treatment, which should be addressed for future app-based studies to achieve long-term effect.

Women’s engagement in the BCS program needs to improve. In our study, the median of usage data showed that the BCS engagement was relatively low, and the big difference between mean and median indicated usage polarization among BCS participants. Meanwhile, our study found that the BCS usage duration was positively related to self-efficacy, social support, and QoL for BCS participants at 3 months, indicating that women drew more benefits if they used the BCS program more often. These usage data could be helpful to explain why the BCS program achieves or fails in the desired outcomes [34]. The design of the BCS program needs to be improved to encourage engagement for a more effective app-based program. In our qualitative process evaluation, women suggested to add message reminders to prompt instant communication and add search engine to help locate information more quickly [15], which could lead to more engagement and should be addressed in the future trial.

Due to time and resource limitations, the participants of this study were recruited from 2 university-affiliated hospitals. Our sample characteristics, such as the participants’ mean age, marital status, educational level, cancer stage at diagnosis, and treatment type were comparable to the national clinical epidemiological data on breast cancer [1,35]. Their current employment status and family income of the participants in this study were also similar to other studies on patients with breast cancer or other cancers during chemotherapy [25,36]. Thus, our study could generalize to women with breast cancer in China with similar characteristics. However, further multicenter studies are needed to provide more conclusive results.

### Limitations

This study possessed several limitations. The requirement of mobile phone internet access may have resulted in a more tech-savvy population who were more comfortable with mobile phone use, potentially limiting the generalization of this study. However, in future, more women will be able to use apps, and the BCS application may be greater. The BCS engagement shows scope of improvement and warrants attention. No long-term effect was found. Future app-based studies should explore different strategies to reduce potential barriers such as the involvement of the caregivers in the app use, to promote engagement by addressing the benefits of this credible resource and health care professionals’ involvement, and to extend the access time to 12 months after the completion of medical treatment to test the long-term follow-up effect. Moreover, the app has not been designed to track women’s usage data on a weekly or monthly basis. The lack of BCS dynamic usage data means that it is not possible to inform how often and how long the BCS program should be used to have a short-term and long-term effect [17]. Continued research is warranted considering the promising findings of this trial.

### Conclusions

The BCS program demonstrates its potential for dissemination globally to support women with breast cancer during chemotherapy. The application of this app seems to be promising for Chinese women with breast cancer in the world. This app also has the potential to be translated to other languages for culturally and linguistically diverse groups. Health care professionals are in a prime position to incorporate app-based program as a routine care to enhance health outcomes for women with breast cancer undergoing chemotherapy, as well as for other cancer patients. This study provides evidence for policy makers and hospital administrators to allocate resources for development and implementation of apps related to health promotion to further advance this effort. This is crucial because mobile apps are being increasingly utilized as supplementary interventions for individuals when the feasibility of face-to-face interventions is challenged by physical limitations or geographic distance [37].

## Appendix

Multimedia Appendix 1 CONSORT-EHEALTH checklist (V 1.6.1).

## Abbreviations

BCS: breast cancer e-support
CAU: care as usual
CHESS: Comprehensive Health Enhancement Support System
FACT-B: functional assessment of cancer treatment-B
HADS: Hospital Anxiety and Depression Scale
HCH: Central South University affiliated Hunan Cancer Hospital
IQR: interquartile range
MDASI: MD Anderson Symptom Inventory
MSPSS: Multidimensional Scale of Perceived Social Support
QoL: quality of life
RCT: randomized controlled trial
SICPA: the Stanford Inventory of Cancer Patient Adjustment
ZSH: Xiamen University affiliated Zhong Shan Hospital
